# Ionic Liquids for Development of Heterogeneous Catalysts Based on Nanomaterials for Biocatalysis

**DOI:** 10.3390/nano11082030

**Published:** 2021-08-10

**Authors:** Anna Wolny, Anna Chrobok

**Affiliations:** Department of Chemical Organic Technology and Petrochemistry, Faculty of Chemistry, Silesian University of Technology, Krzywoustego 4, 44-100 Gliwice, Poland; Anna.Wolny@polsl.pl

**Keywords:** supported ionic liquid phase, supported ionic liquid-like phase, biocatalysis, enzyme, heterogeneous catalysis, immobilization, nanomaterials

## Abstract

The development of effective methods of enzyme stabilization is key for the evolution of biocatalytic processes. An interesting approach combines the stabilization process of proteins in ionic liquids and the immobilization of the active phase on the solid support. As a result, stable, active and heterogeneous biocatalysts are obtained. There are several benefits associated with heterogeneous processes, as easy separation of the biocatalyst from the reaction mixture and the possibility of recycling. Accordingly, this work focused on the supported ionic liquid phases as the efficient enzyme stabilization carriers, and their application in both continuous flow and batch biocatalytic processes.

## 1. Introduction

Increasing the ecological awareness of the society and subsequent restrictive regulations concerning environmental protection, push the chemical industry to develop clean technologies. In order to achieve the United Nations sustainable development goals 2030, each material or product should be safe and sustainable. The use of volatile organic solvents, hazardous substances, production of large amounts of hazardous wastes, and the need to provide large amounts of energy, are the main problems for the environment, associated with chemical processes [1]. Additionally, nanotechnology is often mentioned as a technology that could enable a green growth [2]. Therefore, solutions reducing both the harmful effects of chemical processes on the environment and enhancing synthesis effectiveness are desirable. Green chemistry rules deliver the clues for sustainable and ecological development. One of the most important paths of chemical industry development is the search for a new effective catalysts or/and biocatalyst [3,4].

Enzymes represent a great alternative to conventional catalysts generating hazardous wastes [4]. Enzymes are protein particles that enable various chemical processes to be carried out under mild conditions, and provide high effectiveness of synthesis, due to high enantio-, regio- and chemo-selectivity. Unfortunately, there are some limitations associated with protein applications. Enzymes are sensitive to temperature, pressure, pH changes, and organic solvents. A non-aqueous environment can lead to their folded three-dimensional structure being destroyed, and, in consequence, to biocatalyst deactivation [5]. For that reason, lots of enzymes stabilization methods were developed, e.g., via ionic liquids (ILs), which will be described in this work. Among them, the most common method for enzyme stabilization is immobilization in, or on, a solid matrix. Immobilization not only provides enzyme stabilization, but also enables the easy separation of heterogeneous biocatalysts from the reaction system. The following methods of enzyme immobilization have been developed: entrapment, encapsulation, cross-linking, and adsorption or covalent attachment onto the insoluble carriers [6,7]. Enzyme entrapment and encapsulation are methods that close the protein in a support, without attachment to the matrix. For example, *Candida antarctica* B lipase (CALB) was entrapped in electrospun poly(vinyl alcohol) (PVA) nanofibers. It was reported that entrapped CALB showed higher activity, stability, and reusability, when poly(ethylene glycol) (PEG) was added as additive for lipase immobilization [8]. CALB was encapsulated in the hybrid nanoflowers, consisting of copper (II) or manganese (II) ions, combined with magnetic and carbon nanoparticles. The enzyme performed great catalytic activity, stability, and reusability (eight cycles) in tyrosol ester production [9]. On the other hand, cross-linking is the method for the creation of an intermolecular cross-linkage between the enzyme particles and cross-linking agent, e.g., glutaraldehyde. A special example is the cross-linked enzyme aggregates (CLEAs), which are prepared by using different precipitants (e.g., ammonium sulfate, acetone, PEG) and cross-linkers. Cross-linked CALB aggregates that were obtained with PEG, exhibited the highest activity in the esterification of lauric acid with 1-propanol [10]. CALB was attached onto mesoporous silica nanowires via covalent bonding. Immobilized lipase provided a 94.3% yield (Y) of biodiesel production and a long stability (eight cycles), without a significant loss of activity [11]. The most common among enzyme immobilization methods, is the physical adsorption. This technique is inexpensive, fast, and easy to perform. The activity of the CALB immobilized on various silica supports was studied in the Baeyer–Villiger oxidation of cyclic ketones to lactones. The biocatalysts showed great stability, even in 60% hydrogen peroxide, and enabled high yields of lactones to be achieved [12].

**Figure 1:**
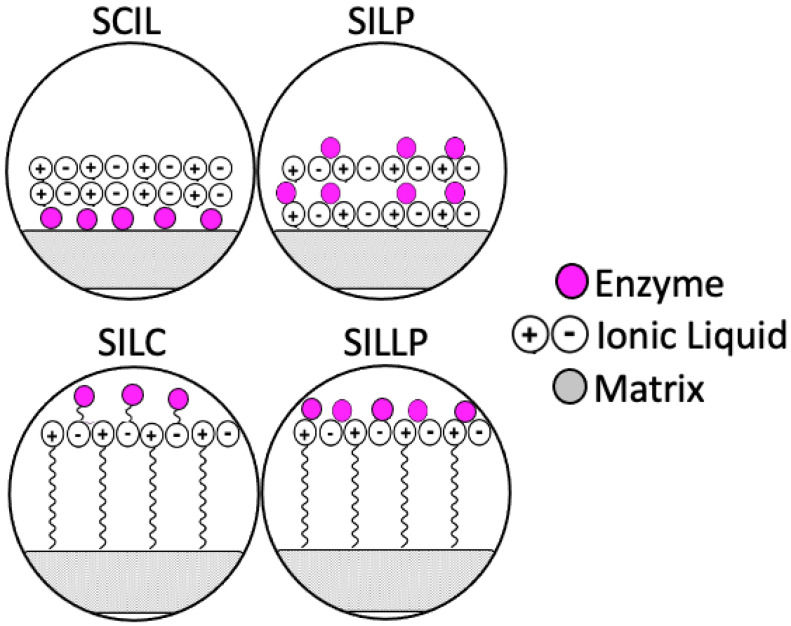
Enzyme immobilized on the supports modified with ionic liquids.

An interesting approach, enhancing the stability and activity of enzymes, proved to be combining ionic liquids and immobilization on solid supports. In this method, the following four different types can be distinguished: supported ionic liquid catalyst (SILC), solid catalyst with ionic liquid layer (SCIL), supported ionic liquid phase (SILP), and supported ionic liquid-like phase (SILLP) (Figure 1).

The supported ionic liquid catalyst technique involves the attachment of an enzyme, via covalent bonding, to ionic liquids moieties that are grafted to the solid matrix. Applications of SILC for enzyme immobilization in biocatalysis were reported. *Candida rugosa* lipase (CRL) was covalently anchored to IL particles that were attached to the magnetic nanoparticles (Fe_2_O_3_). ILs with different chain lengths (C4, C8) and anions (Cl^−^, [BF_4_]^−^, [PF_6_]^−^) were tested both as coupling and stabilizing enzyme agents. The activity of the synthesized SILC biocatalysts was examined in the esters hydrolysis. Covalently immobilized CRL exhibited higher activity and stability than its native form [13]. *Porcine pancreas* lipase (PPL), covalently bonded to alginate nanoparticles, modified with imidazolium ILs, enhanced activity and stability (10 cycles) in triacetin hydrolysis, compared with the free form and immobilized lipase on the magnetic support without IL [14]. IL moieties that were grafted to the magnetic carboxymethyl cellulose nanoparticles, were used in SILC biocatalyst synthesis. The immobilized enzymes PPL and *Penicillin G* acylase activities were tested in triacetin hydrolysis. Both the SILC biocatalysts performed higher activity and stability results than their free forms [15]. *Burkholderia cepacia* lipase (BCL) was covalently anchored to the silica xerogel particles that were treated with protic ionic liquid (PIL), creating an SILC biocatalyst. The synthesized biocatalyst was tested for biodiesel production from different oils (soybean, colza, and sunflower), and provided a conversion between 70–98% [16].

A solid biocatalyst with ionic liquid is the immobilized enzyme on the solid matrix, which is coated with an ionic liquid layer. An example of the SCIL technique in biocatalysis is that CALB immobilized on macroporous acrylic acid beads (Novozyme 435) and coated with 1-butyl-4-methylpyridine hexafluorophosphate [4bmpy][PF_6_], presented an 80% yield of methylglucose fatty acid ester synthesis [17]. Other studies presented Novozyme 435 coated with imidazolium-based ionic liquid, with [PF_6_]^−^ anion, in the ring-opening polymerization (ROP) of lactones. The coated and immobilized lipase showed a two times better conversion of δ-valerolactone than uncoated Novozyme 435 [18]. The same immobilized enzyme coated with imidazolium ILs, with [NTf_2_]^−^ anion, was examined in poly(ε-caprolactone) synthesis. The SCIL biocatalyst provided higher enzyme activity, less IL consumption, higher molecular weight of the polymer and the synthesis yield [19].

The supported ionic liquid phase (SILP) technique consists of the physical immobilization of the enzymatic active phase on the matrix, on which the IL was adsorbed, while the supported ionic liquid-like phase (SILLP) is the method for the physical adsorption of an enzyme on the carrier, which is chemically modified with ionic liquid. The difference in the synthesis of the described carriers is in a different approach, to immobilize an IL on the matrix, for SILP in a physical way and for SILLP through covalent bonding. The applications of SILP and SILLP biocatalysts in biosynthesis, are described, in detail, later in the text. Moreover, other applications of enzymes that are immobilized on SILP and SILLP were also reported. The first example concerns the wastewater treatment. Magnetic chitosan nanoparticles, modified with amino-functionalized ionic liquid, containing 2,2-binamine-di-3-ethylbenzothiazolin-6-sulfonic acid based on copper ion chelation, were used as a carrier for laccase immobilization. The SILLP–laccase system allowed the removal of 2,4-dichlorophenol from water (50 mg/L), with 100% efficiency. SILLP provided higher activity and stability for the enzyme, compared with the free form [20]. SILLPs were also employed as great biosensors. Immobilized cytochrome C on SILLP, based on multiwalled carbon nanotubes (MWCNTs) modified glass carbon electrode, was used in the hydrogen peroxide detection. The investigated biosensor system demonstrated high selectivity, stability, and reproducibility [21]. Peroxidase (PER) from *Pisum sativum* was immobilized on SILP, based on chitin containing gold nanoparticles and used as a biosensor in rosmarinic acid determination. The obtained biosensor presented excellent sensitivity, repeatability, reproducibility, and stability [22]. The next interesting approach is called supported ionic liquid membranes (SILMs). It was reported that enzymatic SILM could be effectively used in the separation of CO_2_ at high temperatures. *Sulfurihydrogenibium yellowstonense carbonic anhydrase SspCA* isozyme was immobilized on SILM made of polyvinylidene fluoride (PVDF), on which [BMIM][NTf_2_] was adsorbed. The thermophilic enzymatic SILM system provided excellent selectivity transport of CO_2_ against N_2_ in high temperatures [23]. Table 1 shows examples of SILC, SCIL, SILP, SILLP techniques that are used in biocatalysis.

To summarize, four different techniques (SILC, SCIL, SILP, and SILLP) of combining ionic liquids and the solid matrix, for enzyme stabilization, were applied in the biocatalysis. Due to the hydrophobic character, ionic liquids that are composed of an imidazolium cation with a long alkyl chain and [NTf_2_]^−^ anion, are the most suitable for enzyme stabilization. The use of hydrophobic ionic liquids enables high specific activities of the enzymes to be obtained, for all types of techniques. It is associated with “essential” water, which is important to the enzymes action. Protein immobilization, via covalent bonding or physical adsorption on the support, ensures high stabilities and activities. The presented techniques also enable easy separation of the biocatalyst from the reaction mixture, and reusability. All these factors allow high conversions, yields, selectivity, and enantioselectivities to be achieved, and make SILC, SCIL, SILP, and SILLP methods attractive for reactions that are catalyzed by the enzymes.

In this paper, the achievements of SILP and SILLP employed in biocatalysis were described. Previously, Domínguez de María et al. referred to supported ionic liquid biocatalysts briefly in 2012 [30]. Next, Lozano et al. reviewed the influence of ionic liquids on enzyme activity, based on the supported ionic liquids in bath and flow biocatalytic processes in 2014 [31]. Again, Lozano et al., in 2015, discussed the supported ionic liquid phases for enzymatic continuous flow synthesis [32]. Afresh, Potdar et al. mentioned about supported ionic liquid phases in the chosen bioprocesses in 2015 [33]. The aim of this article is to complement the time gap, and highlight the strengths and weaknesses of each method, so that synthetic chemists can choose the most suitable method for enzyme immobilization.

## 2. Stabilization of Enzymes via Ionic Liquids

Ionic liquids, known as low-temperature molten salts, are compounds consisting of organic cations and organic or inorganic anions that are classified to green chemistry substances [34]. The lack of volatility, non-flammability, and thermal stability, provide a good alternative as green solvents, which can reduce hazardous organic wastes. The significant characteristic of ionic liquids is the possibility of designing the structure and, as a result, the creation of the unique physicochemical properties [34,35]. Accordingly, ILs have a wide range of industrial applications. Due to their modulated polarity, acidity, and viscosity, ILs are interesting alternatives for conventional organic solvents [36], hazardous catalysts [37], and absorbent and extraction agents [38,39]. Ionic liquids have also been reported as effective enzyme stabilizers [31,40].

In respect to the literature, many methods of enzyme stabilization via ILs were found, which can be classified in two groups.

The first group of methods includes techniques where proteins are modified [30]. Enzyme immobilization is the most common method of enzyme stabilization, it includes sol-gel encapsulation, the cross-linking enzyme aggregates (CLEAs), and the protein attachment to the solid support [31,41]. Sol-gel encapsulation is an irreversible method, where enzymes, via noncovalent interactions, are closed in a support. Encapsulated *Candida rugosa* lipase showed great activities in esterification and hydrolysis reactions. IL was added to the reaction mixture during the immobilization procedure [42]. CLEAs technique is based on the creation of aggregates of enzymes, with the use of glutaraldehyde, which reacts with amino groups in the protein structure. Enzymes are cross-linked in the aqueous solutions with some additives, e.g., ILs. *Burkholderia cepacia* lipase, as CLEA, presented higher catalytic activity than in the native form [43]. The last technique—binding to the solid matrix, via physical adsorption, in the presence of ILs—is known as a supported ionic liquid phases (SILPs) method, and will be described in the next section. Besides the enzyme immobilization technique, there are also methods such as the modification of enzymes with polyethylene glycol (PEG) technique, and propanol-rinsed enzyme preparation (EPRP), but in these cases, ionic liquid is usually used as an additive or solvent [31].

The second category of methods increasing the activity and stability of the enzymes, is environment modification. One of these techniques is the water-in-IL microemulsion (w/IL), which can be described as water droplets, with enzymes dispersed in non-polar ILs. In addition, microemulsion can be stabilized by surfactants, in order to increase the efficiency of emulsifying and protect the protein from denaturation. For example, microemulsion w/IL was tested for *Burkholderia cepacia* lipase in the ester hydrolysis reaction. The catalytic activity of BCL was higher in the w/IL microemulsion than in the w/isooctane microemulsion [44]. Coating proteins with ILs is another method of increasing enzyme stability. Novozyme 435 was coated with ionic liquid and showed higher activity in the synthesis of citronellyl esters than without IL [45].

The modification of the enzyme’s environment can also be assigned the designing of ILs structure, for increasing the enzyme’s activity and stability [30,46,47]. As mentioned before, the structure of ionic liquid can be designed by selecting a proper cation and anion. Different combinations of ions result in the tailoring of the properties of IL, and interactions between IL and enzymes. Main properties of ionic liquid that are important for enzyme stabilization are polarity, anion nucleophilicity, alkyl chain length in the cation, hydrogen bonds, and viscosity [30,31,46,47]. The folded three-dimensional structure of the enzymes can be easily destroyed in non-aqueous media. The “essential” water is needed by the enzymes to save their active conformations, wherefore a little amount of water is usually added to the organic environment [30,31]. Consequently, the activity of the enzymes should be higher in more hydrophobic solvents, because of the lack of affinity to the enzyme’s essential water, which was confirmed by a lot of studies [48,49,50,51]. For example, this was shown by studies on the lipase from *Candida antarctica* activity in butyl butyrate synthesis, via the transesterification reaction of vinyl butyrate and 1-butanol, in the presence of various ionic liquids. CALB exhibited higher activity in more hydrophobic ionic liquids with [NTf_2_]^−^ and [PF_6_]^−^ anions. In this point, the influence of anion nucleophilicity was also explained. The enzyme stability increases with decreasing anion nucleophilicity, due to the more nucleophilic anions that can interact with the positive charges in the enzyme’s structure, and, in the result change, the conformation of the protein. The same studies also confirmed that the lipase stability increases with the length of the alkyl chain in the cation [48]. On the other hand, there are also reports where the enzymes demonstrated better activity in hydrophilic ILs [17,52] or in ILs that consisted of more hydrophobic anions and shorter alkyl chains in the cation [53]. The ability of hydrogen bond forming is the next important factor in enzyme stabilization. It was reported that anions that are hydrogen bond acceptors caused changes in the enzyme’s conformation and denaturation [54,55].

**Figure 2:**
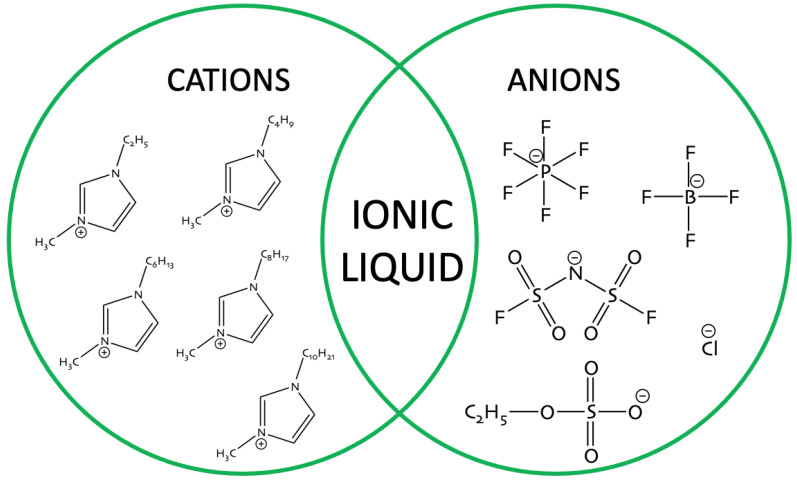
Structures of ionic liquids used for enzyme stabilization.

As has been shown, many factors can affect an enzymes activity and stability. In the designing process of the ionic liquid structure for the biocatalysis process, the nature of the anion, type of cation and the length of alkyl chain, the class of enzyme, and the kind of reaction, should be considered. The most common ionic liquids that are used in biocatalysis, are presented on Figure 2.

## 3. Supported Ionic Liquid Phases in Biocatalysis

**Figure 3:**
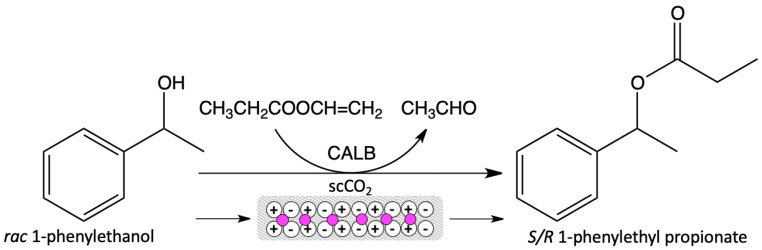
Scheme of kinetic resolution of 1-phenylethanol racemate in SILP biocatalyst and scCO_2_ presence.

The first reports concerning the supported ionic liquid phase (SILP) biocatalysts were reported in 2002. CALB was dissolved in 1-ethyl-3-methylimidazolium triflimide ([EMIM][NTf_2_]) or 1-butyl-3-methylimidazolium triflimide ([BMIM][NTf_2_]), then immobilized on the solid adsorbent (Celite) and used in a continuous butyl butyrate synthesis and the kinetic resolution of 1-phenylethanol in supercritical carbon dioxide (scCO_2_). The scheme of the kinetic resolution of 1-phenylethanol racemate is presented in Figure 3. For both the transesterification reactions, the enzyme showed good catalytic activity, selectivity, and, in the case of the kinetic resolution reaction, high enantioselectivity (>99.9%) and stability (16 cycles) in the anhydrous conditions [24]. In the next year, the same SILP system was tested for the kinetic resolution of 1-phenylethanol at a higher temperature (120 °C) and a lower scCO_2_ pressure (10 MPa). The loss of activity was not observed, even after 10 cycles [25]. In both cases, the use of ionic liquids increased the stability and activity of the enzyme in a high-temperature and scCO_2_ environment [24,25]. Next, the studies of this group focused on the influence of the alkyl chain in the ionic liquid’s cation on the enzyme stabilization, and pointed out the importance of mass transport phenomena between ionic liquid and scCO_2_, in the case of the heterogeneous catalyst. The SILP biocatalyst was tested in the continuous kinetic resolution of 1-phenylethanol in scCO_2_. Immobilized on silica gel, CALB showed a 2000 times longer half-life time in the most hydrophobic IL (hexyl-trimethylammonium bis(trifluoromethylsulfonyl)imide), than in hexane [56]. The SILP-type biocatalyst, in the presence of scCO_2_, was also studied in glycidyl butyrate synthesis. Firstly, the lipases from *Candida antarctica* A (CALA), *Mucor miehei* (MML), and CALB, were mixed with various ionic liquids and then their activity was tested. The CALB in [EMIM][NTf_2_] presented the best R/S ratio. Accordingly, a selected enzyme–IL mixture was immobilized on the solid particles and tested in a continuous process under scCO_2_ conditions, resulting in high enantioselectivity and a little bit lower activity [57].

**Figure 4:**
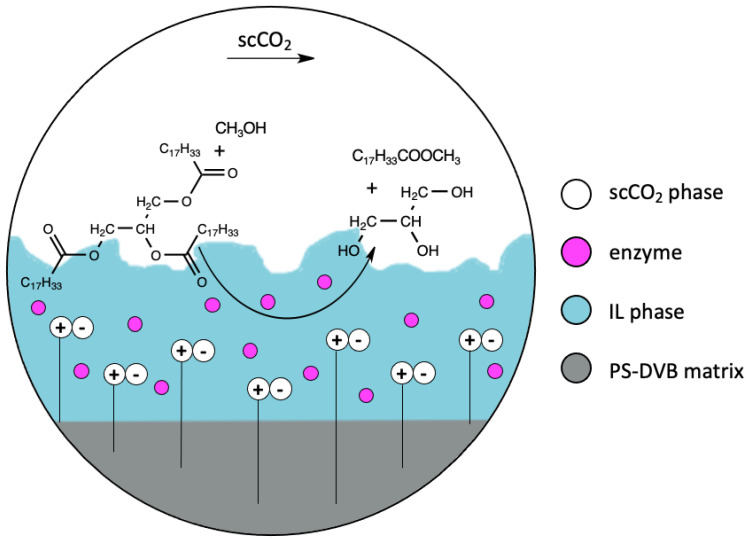
Presentation of enzyme immobilized on SILLP used in methanolysis of triolein under scCO_2_ conditions.

The other approach of performing biocatalysis under scCO_2_ conditions is supported by the ionic liquid-like phase (SILLP). The enzyme–SILLP biocatalysts were described for the first time in 2007. In most cases, the enzymes were immobilized on polystyrene-divinylbenzene (PS-DVB) monolith, modified with imidazolium-based ionic liquids [27]. PS-DVB monolith is a very attractive support, due to the possibility of preparing proper morphologies, shapes, and properties, by cross-linking method synthesis. Modification of the polymeric surface can decrease the hydrophobicity of the support, when it is too high, which, in turn, can protect an enzyme from deactivation [58]. The first-studied SILLP biocatalyst was CALB immobilized onto PS-DVB, modified with imidazolium ionic liquid that was tested in the continuous flow citronellyl propionate synthesis in scCO_2_. A high yield (93%) and stable activity (seven cycles) were reported for that catalytic system, at 80 °C [27]. The same SILLP biocatalyst was used in the combination with zeolites, in the continuous kinetic resolution of 1-phenylethanol in supercritical carbon dioxide. Previous reports of this group resulted in the high technological parameters (*ee* = 97.4%, yield = 74%) when immobilized CALB and zeolites were coated with ionic liquid in that process. To avoid the deactivation of the enzyme, the biocatalyst and acidic catalyst were packed under three different layers [59]. Applying the SILLP biocatalyst in the kinetic resolution process, under scCO_2_ conditions, gave excellent results, such as enantioselectivity >99.9% and a 50% yield (the maximum that is feasible for kinetic resolution). Although, the three-column system, combined with the SILLP biocatalyst–acidic-catalyst–SILLP biocatalyst, resulted in a longer catalyst stability (3 weeks) and higher yield (60%), caused by racemization. Hence, a single column, packed with the mixture of SILLP biocatalyst and zeolite-coated IL, exhibited the best results for the continuous flow kinetic resolution of sec-alcohol (*ee* > 99%, Y = 92%) [60]. The other approach to improve the kinetic resolution of the 1-phenylethanol batch process, involves using the CALB–SILLP biocatalyst under microwave irradiation (MW). The polymeric macroporous PS-DVB support was functionalized in high and low degree. The application of the CALB–SILLP biocatalyst, with a low functionalization degree, containing ionic liquid with [NTf_2_]^−^ anion used in the kinetic resolution process, resulted in high conversion (50%), enantioselectivity (>99%), and stability (12 cycles). The microwave-assisted process showed better performance compared to conventional heating [28]. PS-DVB polymeric monolith, modified with the following ionic liquids: [BMIM][NTf_2_], and 1-octyl-3-methylimidazolium bis(trifluoromethylsulfonyl)imide ([OMIM][NTf_2_]), was synthesized for the immobilization of large biomolecules. Azoalbumin was adsorbed on SILLP, with high efficiency. Moreover, CALB, immobilized on that SILLP, did not show deactivation during the kinetic resolution of 1-phenylethanol, and additionally the activity and the enantioselectivity of this biocatalyst stayed unchanged for 12 months [61]. The polymeric SILLP biocatalyst was also used for biodiesel production, via continuous flow synthesis under scCO_2_ conditions, as presented on Figure 4.

Alkylimidazolium ionic liquid particles were bonded on a PS-DVB support, in different degrees of functionalization. The CALB–SILLP–[NTf_2_]- biocatalyst that was used in the synthesis of methyl oleate, exhibited a yield up to 95% and long stability (45 cycles). Tert-butanol was added in order to prevent the deactivation of the enzyme with the glycerol that was obtained as a by-product [29]. Modified via butylimidazolium, the ionic liquid PS-DVB macroporous support found another one application as a matrix for urease immobilization. SILLP biocatalyst activity was measured for the urea hydrolysis reaction. The activity value was up to 285% higher compared with the native enzyme, and 30 reaction cycles were reported with good activity too [62]. The polymeric PS-DVB matrix was modified in different loading levels of ionic liquids, for dehydrogenase, from *Rhodococcus ruber*, immobilization. Great activity of the biocatalysts was examined in the bioreduction of prochiral ketones to alcohols. Furthermore, enzymatic SILLP material provided high conversion and selectivity values [63].

Besides the PS-DVB matrix, other polymers were also studied as SILP/SILLP supports. A monolithic cellulose-2.5-acetate/polyurethane hybrid, covered with octylmethylimidazolium tetrafluoroborate ([OMIM][BF_4_]), was tested as a hybrid matrix for CALB immobilization. SILP biomaterial was used in the continuous flow transesterification reaction of vinyl-butyrate, and 1-butanol achieved a conversion up to 96% over 18 days [26]. The same hybrid material was used for CALB and CRL immobilization. The SILP bioreactor was tested in a batch and flow kinetic resolution of 1-phenylethanol, synthesis of (−)-2-isopropyl-5-methylcyclohexyl propionate, and in the enantioselective amidation of (*R*,*S*)-1-phenylethylamine with ethyl methoxyacetate. CALB–SILP, consisting of [BMIM][PF_6_] for the transesterification of 1-phenylethanol and vinyl butyrate, under flow and batch conditions, achieved enantioselectivity >99%, and the conversion for batch and continuous processes decreased from 90% to 75%, respectively. CRL–SILP, consisting of [OMIM][PF_6_] in (−)-2-isopropyl-5-methylcyclohexyl propionate production, expressed 79% enantioselectivity, for both the batch and continuous conditions. Amidation of (*R*,*S*)-1-phenylethylamine with ethyl methoxyacetate, catalyzed by CALB–SILP polymeric hybrid, using [BMIM][BF_4_] under flow conditions, resulted in the increase in the enantioselectivity (up to 97%) and activity value compared with the batch process. The SILP hybrid biocatalyst showed great enzyme stability under continuous conditions, at least 7 days for all the described processes, except the esterification reaction, which resulted in the efficient production of enantiomerically pure chemicals technology [64]. The application of cellulose-2.5-acetate beads/hybrid monolith as a CALB–SILP support for the continuous gas-phase transesterification of the vinyl propionate and 2-propanol process, was reported. The best results were obtained with [OMIM][BF_4_]. The enzyme showed excellent stability, even at a high temperature, up to 65 °C over 700 h time on stream [65].

It is worth mentioning another polymeric material that is used as an enzymes SILLP carrier—chitin (poly N-acetylglucosamine) and its derivative chitosan. Chitin and chitosan offer lots of sites that can be functionalized, but these matrixes characterized low porosity and surface area, which reduces the efficiency of the enzyme immobilization. It was reported that ionic liquids can change and modify the morphology of polymeric particles, enabling effective enzyme immobilization [66,67,68]. Studies on the modification of chitin with different ionic liquids were dedicated as SILLP for enzyme stabilization. Various cations, such as (1-methyl-1-propylpyrrolidinium [MPpyrr]^+^, 1-methyl-1-propylpiperidinium [MPpip]^+^, 1,4-dimethyl-1-propylpiperazinium [DMPpz]^+^ and 1-ethyl-3-methylimidazolium [EMIM]^+^_,_ and lactate or acetate anions, were tested for chitin modification. As a result, an SILLP with a large external surface and proper particle sizes, was obtained [66]. Other approach include combined chitosan with mesoporous silica (SBA-15), covalently modified with ionic liquid ([BF_4_]^−^ anion). SILLP nanoparticles were investigated as an excellent support for *Porcine pancreas* lipase (PPL) immobilization, providing high activity and stability (10 cycles) of the enzymes in the triacetin hydrolysis reaction [67]. Magnetic chitosan nanocomposites (chitosan–Fe_3_O_4_), covalently modified by imidazolium ionic liquids with various functional groups, were synthesized as SILLP for PPL immobilization. The enzyme presented a 6.7 times higher activity in triacetin hydrolysis compared to the free form, when IL bonded to magnetic nanocomposites was functionalized with a hydroxyl group with eight carbon atoms, in the imidazolium alkyl chain in the cation of IL [68].

Studies on magnetic supported ionic liquid-type biocatalysts, were reported in 2009. Carriers with magnetic properties can be rapidly and easily separated from the reaction mixture, using an external magnetic field. The modification the magnetic nanoparticles surface is crucial for biosynthesis, due to the changing properties of the support and the increasing stability of the enzymes [69,70,71]. Magnetic nanoparticles (Fe_3_O_4_), combined with silica, covalently modified with imidazolium based ionic liquids with different alkyl chain lengths and anions ([Cl]^−^, [BF_4_]^−^, [PF_6_]^−^), increased the surface area. The activity of CRL, immobilized via physical adsorption, was examined in the esterification of oleic acid and butanol. The enzyme exhibited a 118% higher catalytic activity compared to a native protein, and retained its stability, even at 80 °C [69]. The same SILLP magnetic matrix was applied for *Penicillin G* acylase immobilization. The synthesized carrier provided high enzyme loading and catalytic activity [70]. Another example demonstrated the use of SILLP magnetic bionanoparticles in the production of trans-free plastic fats, via enzymatic interesterifications of solid palm stearin and liquid rice bran oil in a shaking water bath. CRL, immobilized on an Fe_3_O_4_–silica hybrid modified by grafting ionic liquid particles, showed great activity, and provided the synthesis of trans-free plastic fats in the proper composition and desirable physicochemical properties. The lost activity was not reported after the separation of SILLP magnetic nanoparticles, by an external magnetic field [71].

**Figure 5:**
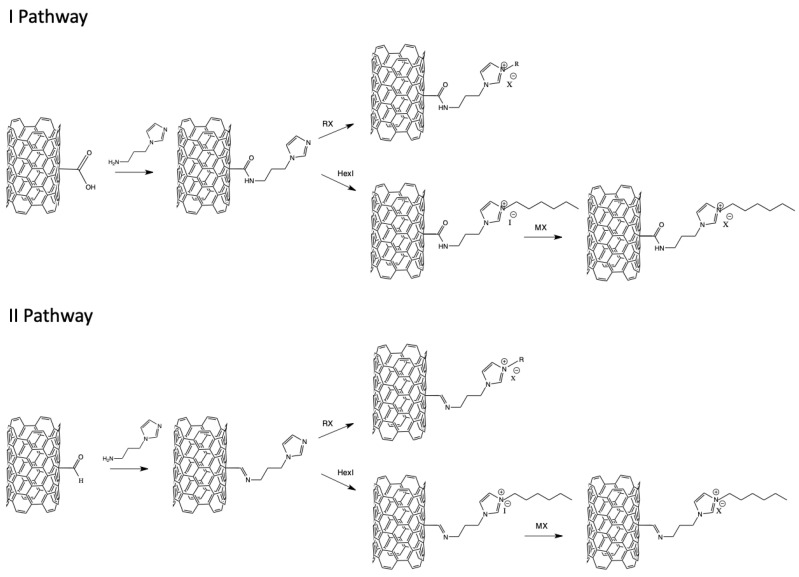
Scheme of MWCNTs chemical modifications with ionic liquids.

Supported ionic liquid phases based on carbon materials, are another significant group of supports in biocatalysis. Active carbon (AC) is an interesting carbon material, with desirable properties for supports in biocatalysis. AC materials are non-toxic, cheap, commercially available, and have a large internal surface area with different sizes of pores, which enables the effective adsorption of enzymes [72]. Lipase PS from *Burkholderia cepacia* was immobilized on an active carbon, active carbon cloth (ACC), activated carbon paper (STV), and alumina, in the presence of ILs—[EMIM][NTf_2_] and [EMIM][BF_4_]. The best results for the enzymatic kinetic resolution of 1-phenylethanol were obtained for lipase immobilized on an active carbon cloth, coated with [EMIM][NTf_2_] in toluene. After 6 h, the achieved product’s enantioselectivity was higher than 99% and the conversion reached 50%, which is the maximum for that reaction type [73]. Another worthwhile carbon material, which can be used as a support, is the carbon nanotube. Carbon nanotubes are an interesting matrix, due to their properties, which include the following: high purity (no possibility of poisoning the enzymatic active phase), mechanical and thermal stability, large surface area, and the possibility of surface functionalization [74]. CALB was immobilized via physical adsorption, on the surface of multiwalled carbon nanotubes (MWCNTs) that were modified with dialkylimidazolium ionic liquids with different lengths of alkyl chains, in combination with [PF_6_]^−^ anion. It was reported that the modification of MWCNTs with ILs did not destroy the support’s structure. The immobilization of an enzyme on the SILLP resulted in higher activity, thermal stability, and longer reusability of the biocatalyst in triacetin hydrolysis compared with CALB adsorbed on MWCNTs, without ILs modification. The longer alkyl chain on IL’s cation shows that the stabilization of the active enzyme’s conformation is more efficient [75]. In the next studies, SILLPs based on MWCNTs were synthesized, as previously described, but the alkyl chain in the imidazolium ring was supplied with different functional groups, such as amino, carboxyl, and hydroxyl. Among them, the CALB–SILLP biocatalyst, functionalized with a hydroxyl group, showed the best results in the triacetin hydrolysis. A selected CALB–SILLP improved activity (18 times compared with CALB–MWCNTs) and thermal stability. Moreover, the biocatalyst retained its stability over four reaction cycles [76]. A further approach considered the selection of an appropriate ionic liquid’s anion for enzyme stabilization. The synthesis pathway described above was used for CALB–SILLP production. The activity of CALB–SILLP biocatalysts with various anions ([Br]^−^, [BF_4_]^−^, [PF_6_]^−^ or [H_2_PO_4_]^−^), were examined in the triacetin hydrolysis. Five times higher activity, compared with CALB–MWCNTs, was demonstrated by CALB–SILLP with [PF_6_]^−^ anion [77]. The next approach employs D-glucose-based ionic liquids that are adsorbed on MWCNTs, for CALB immobilization. The CALB–SILP system was used in the esterification reaction of acrylic acid and n-butanol. The biocatalyst provided a high ester yield (99%), selectivity, and long stability. Lipase retained its activity for five cycles and three cycles, with over a 90% yield of n-butyl acrylate [78]. The next studies of this group included two approaches of the chemical modification of MWCNTs, as presented in Figure 5.

**Figure 6:**
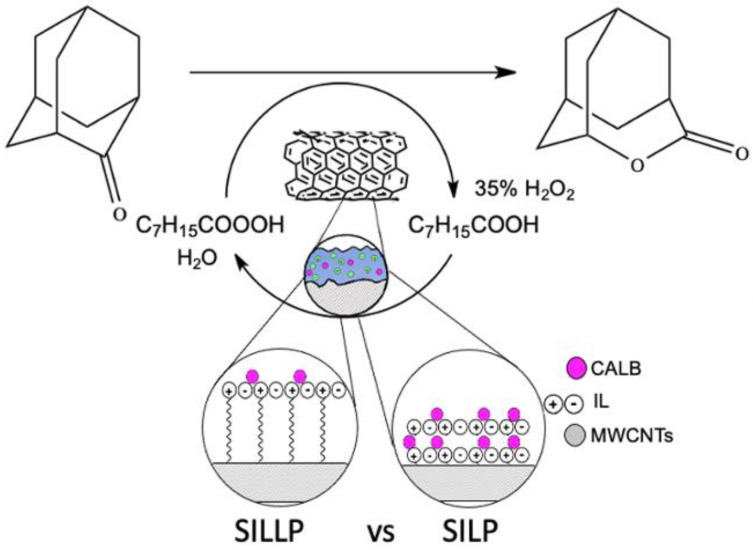
Scheme of Baeyer–Villiger oxidation of 2-adamantanone catalyzed by supported ionic liquid phase biocatalysts.

Ionic liquids were anchored via amide or imine bonds. Prepared SILLPs were tested as carriers for CALB, CRL, and *Aspergillus oryzae* lipase immobilization. Biocatalysts were used in the Baeyer–Villiger oxidation of 2-adamantanone (Figure 6). All the immobilized lipases demonstrated great activity and stability in the aggressive environment of hydrogen peroxide. Four reaction cycles, without a loss of the enzyme’s activity, were performed [79].

Silica materials are the most common group of supports used in SILP and SILLP techniques in biocatalysis. Silica mesoporous materials are characterized by ordered porosity, well-defined pore geometry, large surface area, and possibility of surface modification, which enable high enzyme loading [80]. In the beginning of the section, the first reports about SILP based on silica materials (Celite) were described. The other approach includes supported SILLP for enzyme immobilization. PPL was adsorbed on SILLP, based on mesoporous silica SBA-15. Further, 1-methyl-3-(3-trimethoxysilyl-propyl)imidazolium tetrafluoroborate was attached onto hydroxyl groups on the silica surface via covalent bonding. The enzyme activity in triacetin hydrolysis was improved, from 594 to 975 U/g, after support modification with IL [81]. The next studies of this group focused on the influence of alkyl chain length in the SILLP’ s imidazolium ring on PPL stabilization. Once more, the enzyme showed higher stability and reusability if a longer alkyl chain (C8) was presented in the SILLP structure [82]. Further studies included the effect of various functional groups, such as alkyl, amino, and carboxyl, in the IL structure, attached via covalent bonding onto the mesoporous silica, for PPL activity. Lipase was adsorbed on SILLP and examined in triacetin hydrolysis. Each synthesized SILLP improved protein activity. PPL–SILLP, consisting of IL functionalized with an amino group, expressed the highest activity, which was almost 13 times higher compared with PPL–SBA-15 [83]. Another investigation concentrated on the following different techniques for PPL immobilization: via physical adsorption and covalent bonding. For this purpose, SBA-15 was modified with IL functionalized with a carboxyl group. Both of the biocatalysts presented better stability compared with PPL–SBA-15, but lipase that was grafted into SILLP retained 81.25% of its original activity, when the lipase adsorbed on SILLP retained 52.5% after 20 days of incubation and four reaction cycles [84]. The following experiments checked PLL activity, when the lipase was attached onto the IL anion via ionic binding and cross-linking. The activity of the lipase that was immobilized on the support consisting of imidazolium IL with L-lysine anion, did not decrease after five cycles, so leaching of the enzyme was effectively inhibited [85]. The next studies of this group examined the influence of the particle type grafted into mesoporous silica, for enzyme immobilization and activity in triacetin hydrolysis. The following various supports were prepared: C1–SiO_2_, C8–SiO_2_, C16–SiO_2_, SH–SiO_2_, Ph–SiO_2_, NH_2_–SiO_2_, CH_3_IL–SiO_2_ (methyl group in imidazolium ring), and COOHIL–SiO_2_ (alkyl chain in imidazolium ring functionalized with carboxyl group). The best PPL immobilization efficiency characterized silicas that were modified with ILs, thiol, amino, and phenyl groups. Moreover, PPL–SILLPs exhibited the best thermal stability, pH resistance, and stability among all of the examined biocatalysts. After five cycles, the activity of PPL–SILLP, functionalized with a carboxyl group, was above 62% [86]. A comparison of PLL adsorbed on SILLP, with PLL immobilized via enzyme aggregates, coating onto SBA-15 modified with IL, was also reported. The lipase immobilized as an enzyme aggregate coating demonstrated, after 25 days of storage, 74.25% of its original activity, when PLL–SILLP was only 48% [87]. Further investigations of enhanced enzyme activity led to the increased stability of immobilized PPL via physical adsorption onto SILLP, by embedding it with microspheres of sodium alginate gel. SILLP was obtained by grafting imidazolium-based IL with [BF4]^−^ anion into SBA-15. Novel biocatalysts showed extremely higher thermal stability at 65 °C, almost no activity lost was observed [88]. Papain enzyme was immobilized on SILLP, based on SBA-15. Ionic liquid grafted to the support provided higher papain loading (261 mg/g) than for the enzyme on SBA-15 (185 mg/g). What is more, the papain–SILLP biocatalyst presented higher activity in the case of hydrolysis than papain–SBA-15 [89]. Periodic mesoporous organosilica (PMO) combined with IL was used as a carrier for α-amylase from *Bacillus amyloliquefaciens*. PMO is an organic–inorganic material, where organic groups are ordered inside the silica’s channel walls. Immobilized amylase on PMO-IL retained 88% of its initial activity after four reaction cycles, and showed two times better thermal stability at 70 °C and 80 °C compared with a native form [90]. The activity of CALB was examined in the glycerolysis of corn oil for diacylglycerol production. For that purpose, SBA-15 was modified by grafting onto a hydroxyl group of the imidazolium ring of IL, with two different anions ([BF_4_]^−^, [PF_6_]^−^) and three different alkyl chain lengths (C1, C4, C8) in the cation. The best results were obtained for the CALB–SILLP consisting of a methyl group in the imidazolium ring and [BF_4_]^−^ anion. The activity of CALB–SILLP, in comparison with CALB–SBA-15, increased from 1855 to 5044 U/g. Hence, the enzyme exhibited a greater selectivity towards diacylglycerols synthesis, with the diacylglycerols/monoacylglycerols ratio increasing from 3.72 to 11.99, and the content of the diacylglycerols improving from 53.6 to 67.2%. Also, a retained activity of 90%, after five reaction cycles, for the CALB-SILLP biocatalyst was observed [91]. Another approach uses silica aerogels combined with ILs. BCL lipase was immobilized on the mesoporous silica aerogels that were modified with PIL (PIL–N-methylmonoethanolamine pentanoate—C5) via physical adsorption, covalent bonding, and encapsulation. A comparison of the immobilization yield on PIL-modified support for the performed techniques showed the best results for physical adsorption (83%), and the longest stability presented an encapsulated enzyme (23 reuses) in olive oil hydrolysis as the tested reaction [92]. The same SILLP support was used for BCL immobilization, and applied in ethyl ester production from coconut oil. Further, a 70% conversion was achieved for oils after 144 h at 40 °C (coconut oil:ethanol, 1:7, molar ratio), when BCL–SILLP was used [93]. BCL lipase improved the activity of various phosphonium ILs. Selected trihexyltetradecylphosphonium bis(trifluoromethylsulfonyl)amide [P666(14)][NTf_2_] allowed a relative activity up to 231% and immobilization yield of 98% to be achieved. The biocatalyst kept more than 50% of its initial activity after 26 cycles. It was reported that using [P666(14)][NTf_2_] during the silica support preparation and immobilization procedure significant influences the enzyme activity, stability, and immobilization yield [94].

## 4. Contribution of Nanomaterials to Effectiveness of Supported Ionic Liquid Phases

Nanomaterials are an attractive group of materials for supported ionic liquid phase techniques. Due to their small particle size, nanomaterials offer a larger specific surface area than conventional materials, which can improve the immobilization of the protein’s particles on the support. Moreover, nanomaterials have unique mechanical, thermo-physical properties and surface morphology, which are important in immobilization methods [95,96]. The nanomaterials used in the supported ionic liquid phases, for the biocatalytic reactions described before, are summarized in Table 2.

As shown in Table 2, several groups of nanomaterials that used in the supported ionic liquid phase techniques can be distinguished. Magnetite Fe_3_O_4_ nanoparticles are a very interesting group of nanomaterials, solving problems related to the separation of the nanobiocatalyst from the reaction mixture. The magnetite SILLP biocatalyst can be easily removed by a magnetic field. In the literature, Fe_3_O_4_ nanoparticles were used as a supported ionic liquid biocatalyst, as well as nanocomposites hybrids with chitosan and silica, which prevent the aggregation of magnetite nanoparticles and improve their chemical stability. Additionally, a chemically modified magnetite nanomaterial surface, with hydrophobic ionic liquids, provides high enzyme stability and prevents leaching of the enzyme. Moreover, high ionic liquid loading on magnetite nanomaterials was reported, which can be easily explained by their large surface area [68,69,70,71]. The next significant group of nanomaterials that are used as matrices in SILP/SILLP, is carbon nanotube. Immobilized on carbon nanotubes, lipases exhibit high activity, due to the hydrophobicity of the MWCNTs outermost shells. There are reports where the ionic liquid is covalently grafted, or physically adsorbed, on the MWCNTs surface, which causes the increase in hydrophobicity of the SILP or SILLP matrixes. Therefore, lipases that are immobilized on SILP and SILLP, based on carbon nanotubes, showed great activity and enzyme loading. It is worth emphasizing the interesting approach with the D-glucose-based ionic liquid used for SILP synthesis. In consequence, bio-based SILP was obtained and successfully used in esterification reactions [75,76,77,78,79]. The most widely used in supported ionic liquid phases is silica. The surface of the silica nanomaterials can be easily functionalized. Moreover, the porosity of silica nanoparticles increases ionic liquid loading on the surface, and so on the efficiency of enzyme immobilization. The high activity and thermal stability of the enzymes were observed after immobilization on silica-based SILLP or SILLP [67,81,82,83,84,85,86,87,88,89,90,91,92,93,94].

For all the presented examples, nanomaterials improved the ionic liquid and enzyme loading caused by the large surface area:volume ratio. All of the described nanomaterials can be functionalized, which is key for the supported ionic liquid-like phase method. Nanomaterials based on supported ionic liquid phases, enabled the activity and stability of the employed enzymes to be increased. It was due to the imidazolium ionic liquids containing long alkyl chains and proper anions grafted to the nanomaterial, which increased the hydrophobicity of the support. CALB, PPL, and BCL are the most common lipases used in nanomaterial-based SILP and SILLP. Immobilized enzymes on the nanoparticles that are modified with ionic liquids, catalyzed in hydrolysis, esterification, transesterification and oxidation reactions, provide high yields and conversions. Moreover, biocatalysts could be easy separated from the reaction mixture, especially those with magnetic properties, and used in many cycles.

## 5. Conclusions

In summary, the most important achievements in the use of ionic liquids for the development of heterogeneous catalysts, based on the nanomaterials for biocatalysis, were presented. The environment of the enzyme has a crucial influence on the stabilization of the protein conformation and mass-transport phenomena of products from the support. Ionic liquids play a significant role in the prevention of the enzyme against the destruction of the secondary structure and deactivation under scCO_2_ conditions, high temperatures, and acidic or polar environments. Due to the structural integrity of ionic liquid, support and active conformation of lipase, the activity, stability, and reusability of immobilized enzymes can be improved substantially. The presence of small amounts of water in the IL increases the activity of the lipases. The authors highlighted the importance of the recycling possibility of SILP/SILLP biomaterials. Over the years, various polymer, silica, magnetic and carbon materials were used as matrixes for the supported ionic liquid phase and supported ionic liquid-like phase technique, for enzyme immobilization. The PS-DVB carrier has many advantages, such as the possibility of the design and control the morphology of the material and surface modification, to obtain desired features. It has been reported that chitin and chitosan with low surface areas can be expand via ionic liquid modification. Among the inorganic supports, magnetic nanoparticles have a great potential, because they can be easily separated from the reaction systems by applying an external magnetic field, but the long procedure and low yield of the synthesis decreases their applications. Nanomaterials are significant matrices that provide high ionic liquid and enzyme loading on the surface. Chemical modification of the surface, in comparison with physical adsorption, requires high costs and a long term of preparation, but prevents leaching of the ionic liquid and provides longer reusability. However, for both the SILP and SILLP enzymes, leaching can appear. In all the cases, surface modification of the support, with ionic liquids, is key, because it can make significant improvements in the surface properties, as well as activity and stability of the enzymes. The physical, chemical, and morphological modifications of the support, with ionic liquids, minimize the leaching of the active phase and improve the catalytic activity for continuous and discontinuous processes. The presented reports showed that SILP and SILLP for enzyme immobilization, have a great application potential in biocatalysis.

## Figures and Tables

**Table 1:** Examples of SILC, SCIL, SILP and SILLP applications in biocatalysis.

Type of Technique	Ionic Liquid	Enzyme	Reaction	Ref.
SILC	Imidazolium cation [Cl]^−^ anion	CRL (16 U/mg) ^1^	Esters hydrolysis	[13]
SILC	Imidazolium cation [PF_6_]^−^ anion	PPL (659.4 U/g)	Triacetin hydrolysis	[14]
SILC	Imidazolium cation [PF_6_]^−^ anion	PPL (882.1 U/g)	Triacetin hydrolysis	[15]
SILC	Ammonium cation [C_4_H_9_COO]^−^ anion	BCL (3801 U/g)	Hydrolysis and transesterification of different oils α between 70 and 98% ^2^	[16]
SCIL	Pyridinium cation [PF_6_]^−^ anion	CALB ^3^	Esterification of fatty acids α between 25 and 65%	[17]
SCIL	Imidazolium cation [PF_6_]^−^ anion	CALB ^3^	Ring-opening polymerization of lactone α = 60%	[18]
SCIL	Imidazolium cation [NTf_2_]^−^ anion	CALB ^3^	Ring-opening polymerization of lactone Y = 62% ^4^	[19]
SILP	Imidazolium cation [NTf_2_]^−^ anion	CALB (1.7 U/mg)	Kinetic resolution of 1-phenylethanol via transesterification *ee* > 99.9% ^5^	[24]
SILP	Imidazolium cation [NTf_2_]^−^ anion	CALB (71 U/mg)	Transesterification of vinyl butyrate S = 99% ^6^	[24]
SILP	Imidazolium cation [NTf_2_]^−^ anion	CALB (9.1 U/mg)	Kinetic resolution of 1-phenylethanol via transesterification *ee* > 99.9%	[25]
SILP	Imidazolium cation [BF_4_]^−^ anion	CALB (58 U/mg)	Transesterification of vinyl butyrate α = 96%	[26]
SILLP	Imidazolium cation [Cl]^−^ anion	CALB (20 U/mg)	Transesterification of vinyl propionate Y = 93%	[27]
SILLP	Imidazolium cation [NTf_2_]^−^ anion	CALB (1138.3 U/g)	Kinetic resolution of 1-phenylethanol via transesterification Y = 81%, *ee* = 94%	[28]
SILLP	Imidazolium cation [NTf_2_]^−^ anion	CALB (49.8 U/g)	Triolein transesterification Y = 85%	[29]

^1^ Specific activity. ^2^ Conversion. ^3^ No data available. ^4^ Yield. ^5^ Enantioselectivity. ^6^ Selectivity.

**Table 2:** Nanomaterials used for supported ionic liquid phases in the biocatalysis.

Type	Nanomaterial	Ionic Liquid	Enzyme	Reaction	Ref.
SILLP	Chitosan–silica hybrid	Imidazolium [BF_4_]^−^	PLL (2482 U/g ^1^, 132.1 mg/g ^2^)	Triacetin hydrolysis 35 °C, 10 cycles	[67]
SILLP	Chitosan–Fe_3_O_4_ hybrid	Imidazolium [PF_6_]^−^	PPL (2879 U/g, 118 mg/g)	Triacetin hydrolysis 50 °C, 10 cycles	[68]
SILLP	Fe_3_O_4_	Imidazolium [PF_6_]^−^	CRL (132.3 U/g, 639 mg/g)	Oleic acid esterification 30 °C, 5 cycles	[69]
SILLP	Fe_3_O_4_	Imidazolium [Cl]^−^	*Penicillin G* acylase (261 U/g, 209 mg/g)	Penicillin G potassium salts hydrolysis 37 °C, 10 cycles	[70]
SILLP	Fe_3_O_4_–silica hybrid	Imidazolium [Cl]^−^	CRL	Palm stearin interesterification 45 °C, 4 cycles	[71]
SILLP	MWCNTs	Imidazolium [PF_6_]^−^	CALB (19,354 U/g, 96 mg/g)	Triacetin hydrolysis 60 °C, 4 cycles	[75]
SILLP	MWCNTs	Imidazolium [PF_6_]^−^	CALB (25,350 U/g, 114 mg/g)	Triacetin hydrolysis 60 °C, 4 cycles	[76]
SILLP	MWCNTs	Imidazolium [PF_6_]^−^	CALB (13,636 U/g, 66 mg/g)	Triacetin hydrolysis 60 °C, 4 cycles	[77]
SILP	MWCNTs	D-glucose based [NTf_2_]^−^	CALB (42 mg/g)	Acrylic acid esterification 25 °C, 5 cycles, Y = 99% ^3^	[78]
SILLP	MWCNTs	Imidazolium [Oc_2_PO_4_]^−^	CALB (64 mg/g)	2-adamantanone oxidation 20 °C, 5 cycles, α = 91% ^4^	[79]
SILP	MWCNTs	Imidazolium [NTf_2_]^−^	CALB (22 mg/g)	2-adamantanone oxidation 20 °C, 4 cycles, α = 99%	[79]
SILLP	Silica	Imidazolium [BF_4_]^−^	PPL (975 U/mg)	Triacetin hydrolysis 36 °C, 5 cycles	[81]
SILLP	Silica	Imidazolium [BF_4_]^−^	PPL (975 U/mg)	Triacetin hydrolysis 35 °C, 5 cycles	[82]
SILLP	Silica	Imidazolium [BF_4_]^−^	BCL (10205 U/g, 230 mg/g)	Triacetin hydrolysis 50 °C, 3 cycles	[83]
SILLP	Silica	Imidazolium [BF_4_]^−^	PPL (720 U/g, 227.5 mg/g)	Triacetin hydrolysis 35 °C, 4 cycles	[84]
SILLP	Silica	Imidazolium L-lysine	PPL (244 U/g, 197 mg/g)	Triacetin hydrolysis 50 °C, 5 cycles	[85]
SILLP	Silica	Imidazolium [BF_4_]^−^	PPL (392 U/g, 245 mg/g)	Triacetin hydrolysis 50 °C, 5 cycles	[86]
SILLP	Silica	Imidazolium [BF_4_]^−^	PPL (760 U/g, 117 mg/g)	Triacetin hydrolysis 45 °C, 5 cycles	[87]
SILLP	Silica	Imidazolium [BF_4_]^−^	PPL (468 U/g, 186 mg/g)	Triacetin hydrolysis 45 °C, 5 cycles	[88]
SILLP	Silica	Imidazolium [Cl]^−^	Papain (0.8 U/mg, 261 mg/g)	L-tyrosine synthesis 50 °C	[89]
SILLP	Organosilica	Imidazolium [Cl]^−^	Amylase from *Bacillus amyloliquefaciens* (29.35 U/mg, 80 mg/g)	Starch hydrolysis 70 °C, 4 cycles	[90]
SILLP	Silica	Imidazolium [BF_4_]^−^	CALB (5044.44 U/g)	Corn oil glycerolysis 50 °C, 5 cycles, α = 70.94%	[91]
SILP	Silica aerogel	Ammonium [C_4_H_9_COO]^−^	BCL (83% ^5^)	Olive oil hydrolysis 37 °C, 23 cycles	[92]
SILP	Silica aerogel	Ammonium [C_4_H_9_COO]^−^	BCL (337 mg/g)	Coconut oil esterification 40 °C, α = 70%	[93]
SILP	Silica	Phosphonium [NTf_2_]^−^	BCL (91.1%)	Olive oil hydrolysis 37 °C, 17 cycles	[94]

^1^ Specific activity of enzyme. ^2^ Enzyme loading on the support. ^3^ Yield. ^4^ Conversion. ^5^ The total activity recovery yield.

## Data Availability

Data sharing is not applicable for this article.
